# Playability of school-environments and after-school physical activity among 8–11 year-old children: specificity of time and place

**DOI:** 10.1186/s12966-016-0407-5

**Published:** 2016-07-15

**Authors:** Teun Remmers, Dave Van Kann, Carel Thijs, Sanne de Vries, Stef Kremers

**Affiliations:** Department of Epidemiology, Maastricht University (Medical Center+), CAPHRI School for Public Health and Primary Care, P.O. Box 616, 6200MD Maastricht, The Netherlands; Department of Health Promotion, Maastricht University (Medical Center+), CAPHRI School for Public Health and Primary Care, Maastricht, The Netherlands; The Hague University of Applied Sciences, Research group Healthy Lifestyle in a Supporting Environment, The Hague, The Netherlands; Department of Health Promotion, Maastricht University (Medical Center+), NUTRIM School for Nutrition and Translational Research in Metabolism, Maastricht, The Netherlands

**Keywords:** Accelerometer, School, Child, Environment, Distance, Time specific, Methodology, GIS, Geospatial information, Audit

## Abstract

**Background:**

Physical Activity (PA) occurs in several behavioral domains (e.g., sports, active transport), and is affected by distinct environmental factors. By filtering objective PA using children’s school schedules, daily PA can be separated into more conceptually meaningful domains. We used an ecological design to investigate associations between “playability” of 21 school-environments and children’s objectively measured after-school PA. We also examined to what extent distinct time-periods after-school and the distance from children’s residence to their school influenced this association.

**Methods:**

PA was measured in 587 8–11 year-old children by accelerometers, and separated in four two-hour time-periods after-school. For each school-environment, standardized playability-scores were calculated based on standardized audits within 800 m network buffers around each school. Schools and children’s residences were geocoded, and we classified each child to be residing in 400, 800, 1600, or >1600 m crow-fly buffers from their school. The influence of network-distance buffers was also examined using the same approach.

**Results:**

Playability was associated with light PA and moderate-to-vigorous PA after-school, especially in the time-period directly after-school and among children who lived within 800 m from their school. Playability explained approximately 30 % of the after-school PA variance between schools. Greater distance from children’s residence to their school weakened the association between playability of the school-environments and after-school PA.

**Conclusions:**

This study demonstrated that relationships between the conceptually matched physical environment and PA can be revealed and made plausible with increasing specificity in time and distance.

## Background

The short- and long-term benefits of physical activity (PA) in children are well known. The role of attributes of the physical environment in regulation of children’s PA behavior has been given increasing attention in recent years, but results so far have been mixed [1]. Although the use of objective measurements is preferred in PA-related research involving children (e.g., by accelerometers), investigating relationships between PA and the physical environment using objective measurements proves to be challenging [2].

A first challenge is assessing children’s exposure to detailed elements of the physical environment. Researchers in the disciplines of health sciences, urban planning, and leisure studies all contribute to the development of measurements assessing these environmental elements [3]. In general, three types of measurements can be identified; self-administered surveys, systematically completed audits, and GIS-based measures [4]. In terms of objective measurements, GIS-based measures may currently be more suitable for assessing design-related features of neighborhoods on a larger geographic scale. Audits, in turn, may be more suitable to assess qualities of environmental elements in smaller-scaled environmental settings [4, 5]. In studies investigating PA in children, audits may thus be favorable in detecting (quality of) small-scaled environmental opportunities that may potentially influence leisure time PA (e.g., attractiveness and quality of public spaces or playgrounds). Recently, an instrument assessing detailed playground characteristics using systematic in-person audits of environments have been introduced as a “playability index” [6]. This index stems from the Environmental Assessment of Public Recreational Spaces (EAPRS) [7] and assesses qualities of playground-features such as facilities, aesthetics, proximity, and accessibility.

A second challenge when investigating relationships between PA and the physical environment stems from the paradigm that PA occurs in several conceptual domains (i.e., leisure, school, transport and home) [8–10]. Investigating associations between the environment and overall PA may lead to inconsistencies, as different PA domains are regulated by distinct environmental factors [4]. An example of domain-specificity relates to children’s school schedule, which largely limits their spatial freedom- and thus environmental exposure during weekdays. Separately investigating after-school PA (ASPA) helps to increase our understanding children’s context-specific PA and its environmental attributes [2, 9, 11–13]. Studies using subjective measures of ASPA generally reported that boys seemed to be more active after school than girls [14, 15] and suggested a negative influence of technology-related sedentary activities on ASPA [15, 16]. The studies that used objective measures generally indicated that ASPA contributed considerably to total PA, that boys were indeed more active after school [17–27]. More specifically, one study reported that children do not compensate inactive days at school by increasing ASPA on a weekly basis [28]. Three studies reported on relationships between ASPA and objectively audited features of the environment [22, 24, 26]. Results generally revealed that time outside resulted in 2–3 fold higher ASPA [26], but no associations were found between the number and proximity of PA- facilities / public open spaces / playgrounds in the environment (and their specific features such as lightning and trees) and ASPA in children [22, 24]. However in the audits of the studies above, no information was recorded about the quality of these PA-facilities (e.g., attractiveness, maintenance status or age-appropriateness). This may be important, as other factors than actual distance to public open spaces may determine the use of public open spaces or playgrounds [24, 29].

An advantage of investigating ASPA is that when using exact school bell-times, relationships between ASPA and attributes of the school-environment can be investigated with an equal starting-point; both regarding time-opportunities and geographical location for all children attending the same school. To even further improve our understanding of this association, the ASPA time-period may be separated into even more precise time-segments after school bell-times. For example, by theory, children are all optimally exposed to the school-environment directly after school ends (i.e., bell times) but to a lesser extent later in the afternoon. Greater distances of a child’s residence to the school-environment may attenuate relationships between playability and ASPA, because children living further away may be more likely to engage in ASPA at places outside the school-environment under study.

Consequently, the present study used an ecological approach to investigate the association between environmental playability and objectively measured ASPA of 8–11 year-old children, using audits of school-environments. In addition, we aimed to demonstrate that with increasing specificity in time and distance, relationships between school-environments and ASPA can be revealed and made plausible.

## Methods

This investigation was embedded in a prospective study in the Southeast part of the Netherlands, focusing on environmental attributes and PA in Dutch primary school children. The design and protocol are described in detail elsewhere [30]. After obtaining parental informed consent, 815 sixth and seventh grade primary-school pupils from 21 schools participated in PA measurements and questionnaires for both one of the parents and child. Data collection took place between the 26^th^ of September and the 1^st^ of December 2012 and analyses were performed in 2015. The Medical Ethics Committee of the Maastricht University Medical Center approved this study (reference number METC 12-4-077).

### Measurements

#### After-school physical activity

ASPA was measured using ActiGraph GT3X+ accelerometers (30Hz) for five consecutive days (ActiGraph, Pensacola, FL), defining non-wear periods according to 60 min of consecutive zero’s according to Troiano’s criteria [31]. Activity intensity classification was based on Evenson’s cutpoints [32]. Participants were instructed to only remove the accelerometer in water-related activities, so we explicitly instructed them to keep wearing them during sports-activities. We excluded measurements containing less than 250 min per day of registration time, for at least two weekdays. Although studies investigating whole-day PA patterns usually apply more stringent criteria for these registration times [33], we were only interested in a smaller part of the daily PA pattern and therefore required less registration time. Weekend days and Wednesdays (because of a shortened school-schedule) were excluded. Accelerometry was aggregated to hourly averages, for each day of measurement. Using weartime-filters, ASPA was filtered from total PA registration time, based on exact school’s bell times. ASPA was then separated in four two-hour time periods: 1) directly after-school–16:00, 2) 16:00–18:00, 3) 18:00–20:00, and 4) 20:00–22:00. All schools ended between 14:45 and 15:30. To ensure that these time-periods represented hourly patterns, and are thus not influenced by spurious PA-spikes in children with limited period-specific weartimes, we only included accelerometer data that consisted of at least 50 % of the period-specific registration time (i.e., at least one hour in a two-hour registration period) [26, 27, 34]. For the first time period, we tailored the percentage of period-specific weartime based on individual school bell-times.

Based on data from the Royal Dutch Meteorological Institute (KNMI), we also identified meteorological circumstances (i.e., average temperature, average duration of rainfall, and average duration of sunshine per day) during measurement-days.

#### Playability

Playability of the school-environments was assessed by two trained researchers using the SPACE observation instrument [35, 36], within an 800 m radius from each school, while acknowledging natural barriers such as highways or canals. This 54-item instrument audits PA friendliness of neighborhoods and assesses characteristics such as residential density, playground characteristics, and traffic intensity, based on the Neighborhood Environment Walkability Scale but modified to reflect the Dutch environmental context [37, 38]. Inter-rater agreement between the two researchers who audited school-environments was acceptable (Kappa = 0.73). Playability was operationalized by first extracting items representing characteristics of playgrounds (excluding schoolyards) within the 800 m crow-fly surface areas. Extracted were the playground’s size in squared meters, accessibility (safely accessible versus not-safely accessible), opening hours (unlimited versus limited), maintenance-status (poor versus good), number of facilities (e.g., climbing-facilities and soccer goals), and age-appropriateness of these facilities for 8–11 year-old children (none, partly, and fully age-appropriate). Each individual playground-characteristic was summed and standardized based on equal weights, to reflect one standardized score for each individual playground. Subsequently, these scores were aggregated to a playability index-score for each school-environment.

#### Distance from children’s residence to their school

**Fig. 1:**
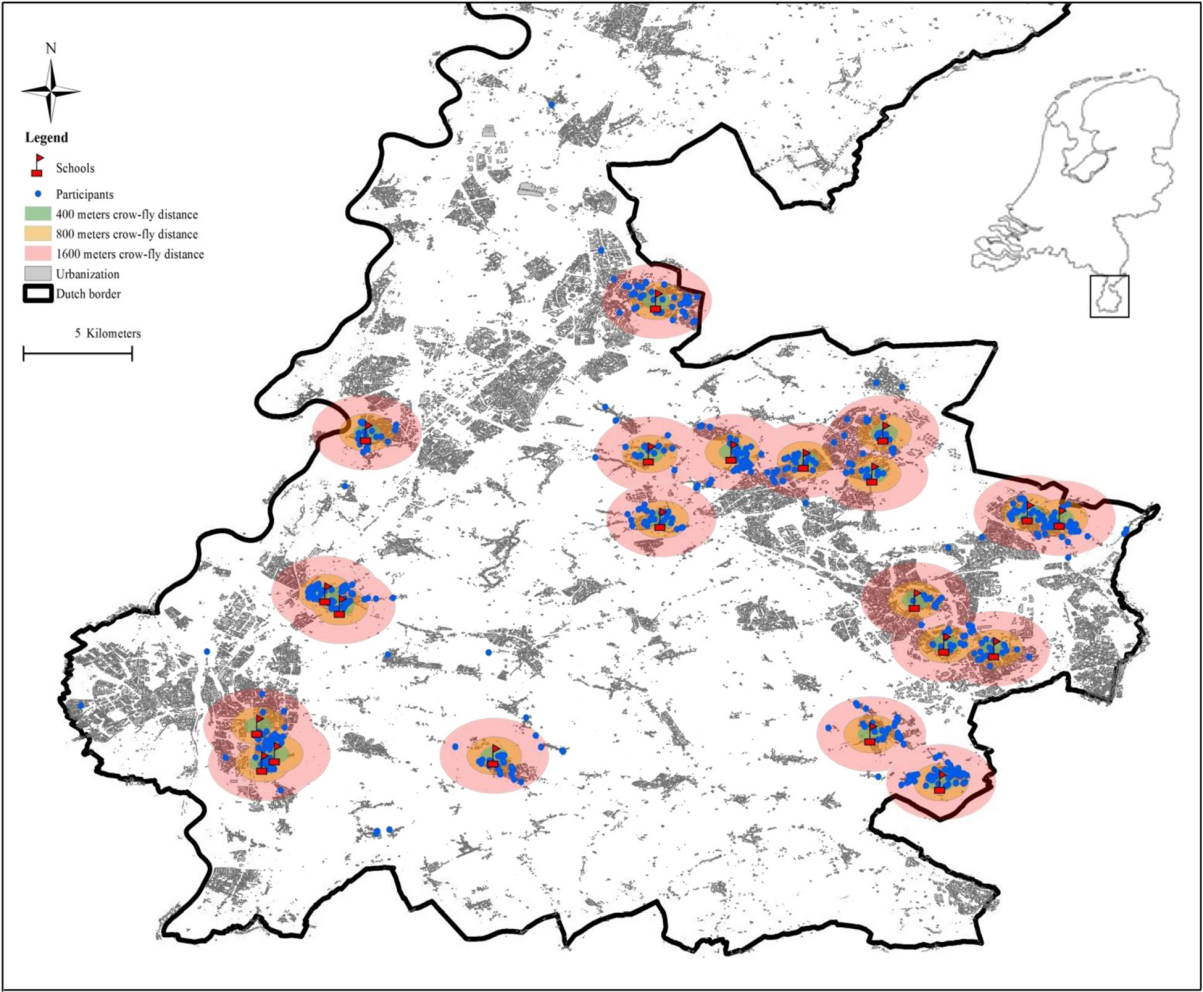
Geographical location of included schools and participants

Since in the Netherlands a limited number of primary schools generally cover a small residential area, parent’s decisions regarding the school of their children is often based on the (close) distance from their residence [39, 40]. Because of this vicinity to their school, children from the same school share large parts of their physical environment (Fig. 1). Therefore, these shared school-environments provide unique opportunities for investigating relationships between ASPA and the physical environment. Location of schools and respondent’s residences were geocoded, and we computed a 400, 800 and 1600 m crow-fly buffer around each school using ArcGIS (ESRI ArcGis Desktop 10.2. Redlands, CA). We subsequently classified each residence to be located 1) inside the 400 m buffer-area, 2) outside 400, but inside 800 m, 3) outside 800, but inside 1600 m, 4) outside the 1600 m buffer area. As crow-fly distances may be misleading because of barriers in the environment (e.g., highways or canals), we also computed network-distance as the shortest network distance in meters via the street network from each child’s residence to their school using Google Maps (GoogleMaps, 2015), and recoded distances in four categories. In order to keep sample sizes within categories comparable with the crow-fly distance, we based categorization on equal frequency distributions.

### Statistical analyses

Our analyses were performed using SPSS 20.0 for Windows (IBM SPSS Inc., Armonk, NY), and *p* <0.05 indicated statistical significance. Our dependent variable was the ASPA performed in light and moderate to vigorous intensity for each hourly time-interval of measurement. Our primary independent variable was the combined playability index of the different school-environments. We first described the percentages of light PA (LPA) and moderate-to-vigorous PA (MVPA) across four two-hour time-periods after school, and across four distance-buffers from schools using univariate analyses of variance (Table 1).

**Table 1:** Descriptive statistics of the study population

Individual level (*n* = 587)	
Age; mean years (sd) (missing *n* = 10)	10.2 (0.7)
Gender; *n* boys (%)	280 (47.7)
Ethnicity *n* Dutch (%) (missing *n* = 9)	490 (84.4)
Crow-fly distance from home to school; *n* (%)	
within 400 m	187 (31.9)
within 800 m	225 (38.3)
within 1600 m	119 (20.3)
outside 1600 m	56 (9.5)
Network distance from home to school; *n* (%)	
distance ≤ 499 m	138 (23.5)
distance 500–799 m	137 (23.3)
distance 800–1199 m	171 (29.1)
distance ≥ 1200 m	141 (24.0)
Light PA by time of the day; mean % of time per day (sd)	27.9 (6.6)
end of the school day – 16:00 h; mean % (sd) (*n =* 539)	35.3 (8.8)
16:00–18:00 h; mean % (sd) (*n =* 586)	29.2 (8.2)
18:00–20:00 h; mean % (sd) (*n =* 585)	27.5 (7.7)
20:00–22:00 h; mean % (sd) (*n =* 326)	20.8 (9.5)
MVPA by time of the day; mean % of time per day (sd)	7.7 (4.0)
end of the school day – 16:00 h; mean % (sd) (*n =* 539)	10.4 (6.2)
16:00–18:00 h; mean % (sd) (*n =* 586)	6.8 (4.8)
18:00–20:00 h; mean % (sd) (*n =* 585)	9.2 (7.5)
20:00–22:00 h; mean % (sd) (*n =* 326)	4.8 (6.2)
Light PA by crow-fly distance; mean % of time per day (sd)	27.9 (6.6)
within 400 m (*n =* 187)	28.2 (7.0)
within 800 m (*n =* 225)	28.0 (6.4)
within 1600 m (*n =* 119)	27.9 (6.3)
outside 1600 m (*n =* 56)	27.3 (6.5)
MVPA by crow-fly distance; mean % of time per day (sd)	7.7 (4.0)
within 400 m (*n =* 187)	7.9 (4.1)
within 800 m (*n =* 225)	7.9 (4.1)
within 1600 m (*n =* 119)	7.4 (4.0)
outside 1600 m (*n =* 56)	7.3 (3.8)

We performed multilevel linear mixed models in order to account for the time-dependent structure of the data. We specified a random intercept and slope for the hourly time-intervals, nested within the specific dates at which a respondent’s accelerometry commenced. Analyses were also adjusted for hourly weartime, average daily temperature, daily duration of rainfall, and daily duration of sunshine per day. We evaluated whether age and gender moderated the association between playability and ASPA, but as we did not find moderation, we only adjusted for age and gender.

To investigate the influence of the four time-periods and distance-categories on the relationship between ASPA and playability, we entered the appropriate interaction terms in our linear mixed models. Using dummy-coded interaction-terms between distance-categories and the playability index-score, we were able to estimate main effects of playability for each of the two-hour time periods, while still acknowledging the time-dependent structure with the random intercept and slope. Distance-categories were both conceptualized as crow-fly and network-distances. Finally, we also repeated our analyses now stratifying for the time-periods and distance-categories simultaneously to investigate their interactive influence (Table 4).

## Results

In total, 587 children (74.2 %) provided valid ASPA measurements, for two (44.8 %) and three (55.2 %) valid weekdays, respectively. The 280 participating boys and 307 girls were aged 10.2 years on average (range 8 to 11 years) (Table 1). Across all time-periods, 27.9 % of the time after-school was spent in LPA, which accumulated to 103.4 min per day (SD = 23.9). MVPA accounted for 7.7 % of the after-school time, accumulating to 28.4 min per day (SD = 14.6). Daily percentages of LPA declined across the four time-periods, while MVPA slightly declined after 16:00, but increased again after 18:00. Daily percentages of LPA and MVPA were comparable across the distance-categories (no statistically significant differences in analysis of variance, data not shown).

### Playability and after-school Physical Activity by time-period

Children who attended schools in areas with higher playability scores, generally showed significantly higher LPA and MVPA in the first two time-periods (i.e., between the end of the school day and 18:00) (Table 2). By contrast, in the subsequent time-periods, the association between playability and ASPA attenuated. Only the relatively small amount of time performed in light intensity between 20:00 and 22:00 unexpectedly showed a statistical significant relationship with playability.

**Table 2:** Associations between playability and after-school physical activity intensities by time of the day

	% Light PA	% MVPA
Time of the day		
End of the school day – 16:00 h (*n =* 539)	**0.043 (0.026 to 0.060)**	**0.029 (0.008 to 0.048)**
16:00–18:00 h (*n =* 586)	**0.018 (0.002 to 0.034)**	**0.028 (0.012 to 0.045)**
18:00–20:00 h (*n =* 585)	0.001 (−0.015 to 0.018)	−0.007 (−0.024 to 0.010)
20:00–22:00 h (*n =* 326)	**0.052 (0.032 to 0.072)**	0.016 (−0.005 to 0.038)

Standardized beta’s (with 95 % confidence intervals in brackets) from linear mixed model analyses with a random intercept and slope over time (one-hour periods), nested within the dates at which measurement commenced. Results were adjusted for age, gender, average temperature, average duration of rainfall, and average duration of sunshine per day. Bold number represents statistical significance at *p* < 0.05

### Playability and after-school Physical Activity by distance-categories

Only if children lived within 400 m from their school, after school LPA was positively associated with playability (Table 3). When children lived beyond 400 m from their school, associations attenuated and were no longer statistically significant. For after school MVPA, the attenuating influence of distance from home to school was detectable when exceeding the 800 m crow-fly or network-distance.

**Table 3:** Associations between playability and after-school physical activity, stratified by distance from children’s residence to school

% Light PA				
Crow-fly distance from children’s residence to school	<400 m	400–800 m	800 - 1600 m	outside 1600 m
	**0.039 (0.001 to 0.067)**	0.018 (−0.007 to 0.042)	0.014 (−0.015 to 0.044)	0.007 (−0.039 to 0.053)
Network-distance from children’s residence to school	≤ 499 m	500–799 m	800–1199 m	≥ 1200 m
	**0.040 (0.007 to 0.071)**	0.020 (−0.011 to 0.052)	0.023 (−0.004 to 0.049)	0.004 (−0.026 to 0.034)
% MVPA				
Crow-fly distance from children’s residence to school	within 400 m	within 800 m	within 1600 m	outside 1600+ meters
	**0.026 (0.004 to 0.049)**	**0.038 (0.018 to 0.057)**	0.018 (−0.006 to 0.041)	−0.005 (−0.042 to 0.031)
Network-distance from children’s residence to school	≤ 499 m	500–799 m	800–1199 m	≥ 1200 m
	**0.027 (0.002 to 0.052)**	**0.046 (0.021 to 0.071)**	0.020 (−0.001 to 0.041)	0.011 (−0.013 to 0.035)

Standardized beta’s (with 95 % confidence intervals in brackets) from linear mixed model analyses with a random intercept and slope over time (one-hour periods), nested within the dates at which measurement commenced. Results were adjusted for age, gender, average temperature, average duration of rainfall, and average duration of sunshine per day. Bold number represents statistical significance at *p* < 0.05

To examine the interactive influence of time and distance, analyses of time-periods were stratified for distance-categories from home to school (Table 4). The results of these stratified analyses were generally in line with the results described above. Regarding LPA, the attenuating influence of time-periods and distance-categories was comparable to the results Tables 2 and 3, except for the distance greater than 1600 m and time-period after 20:00 h. Regarding MVPA, attenuating influences of time and distance were also comparable with Tables 2 and 3, but in some instances revealed deeper insights. For example, ASPA after 16:00 h (i.e., second time-period) was only associated with playability in children that lived within 400 m from their school. When these analyses were repeated using network-distance, similar attenuating influences of time and distance were found (Appendix: Table 5).

**Table 4:** Associations between playability and after-school physical activity, stratified for time of the day and crow-fly distance from children’s residence to school

	% Light PA			
	Crow-fly distance from children’s residence to school			
Time of the day	within 400 m	within 800 m	within 1600 m	outside 1600+ meters
End of the school day – 16:00 h (*n =* 539)	**0.065 (0.032 to 0.100)**	**0.046 (0.015 to 0.074)**	**0.045 (0.009 to 0.080)**	0.001 (−0.052 to 0.054)
16:00–18:00 h (*n =* 586)	0.027 (−0.004 to 0.058)	0.013 (−0.012 to 0.039)	0.031 (−0.0001 to 0.062)	0.009 (−0.041 to 0.059)
18:00–20:00 h (*n =* 585)	0.024 (−0.008 to 0.057)	0.014 (−0.013 to 0.041)	−0.022 (−0.055 to 0.010)	−0.021 (−0.074 to 0.032)
20:00–22:00 h (*n =* 326)	**0.051 (0.012 to 0.090)**	**0.061 (0.028 to 0.093)**	**0.043 (0.004 to 0.081)**	**0.075 (0.011 0.140)**
	% MVPA			
	within 400 m	within 800 m	within 1600 m	outside 1600+ meters
End of the school day – 16:00 h (*n =* 539)	**0.047 (0.010 to 0.083)**	**0.046 (0.012 to 0.080)**	−0.010 (−0.051 to 0.031)	0.006 (−0.053 to 0.065)
16:00–18:00 h (*n =* 586)	**0.042 (0.010 to 0.074)**	0.023 (−0.005 to 0.050)	0.034 (−0.0003 to 0.068)	−0.006 (−0.057 to 0.046)
18:00–20:00 h (*n =* 585)	0.017 (−0.016 to 0.050)	0.008 (−0.020 to 0.037)	−0.027 (−0.063 to 0.008)	**−0.088 (−0.141 to−0.034)**
20:00–22:00 h (*n =* 326)	−0.06 (−0.046 to 0.034)	0.013 (−0.023 to 0.048)	0.017 (−0.025 to 0.060)	**0.100 (0.030 to 0.168)**

Standardized beta’s (with 95 % confidence intervals in brackets) from linear mixed model analyses with a random intercept and slope over time (one-hour periods), nested within the dates at which measurement commenced. Results were adjusted for age, gender, average temperature, average duration of rainfall, and average duration of sunshine per day. Bold number represents statistical significance at *p* < 0.05. Network distance showed comparable results: Appendix: Table 5

## Discussion

This study investigated the association between playability of school-environments and ASPA, separately for time-periods within the after-school period and distance-categories from school to children’s residence. We demonstrated that the influence of playability is highly dependent on these time-periods and distance from home to their school: greater distance attenuated the influence of playability of the school-environment on ASPA, especially in the first hours after-school. As expected, children that lived outside the study-area for which playability was audited generally showed no relationships between playability of the school-environment and ASPA.

When comparing our findings in the light of other studies that have investigated ASPA objectively, we can confirm that boys were more active after-school compared to girls [17, 20, 25]. In contrast to the study of Mota et al., which reported that boys were more active in the later time-periods after school [19], we found that the difference between boys and girls was stable across the first three time-segments, and this difference decreased at later periods in the evenings (data not presented). As this study did not compare PA during school hours with ASPA, we cannot compare our results with studies that indicated that ASPA significantly contributed to total PA [17, 20, 21]. Although Timperio et al. found that relationships between ASPA and individual features of public open spaces were different for boys than for girls [22], we found no such moderation mechanisms in our playability index. This may be because in our study, potential gender differences may annul at a higher level of abstraction when utilizing a standardized index-score of playground qualities instead of individual features of public open spaces. Further research is however needed to clarify potential gender-related moderation mechanisms. In addition, Scott et al., argued that perceptions of easy access and the number of PA-facilities, but not objectively determined number and proximity of PA-facilities were related to adolescent girls’ non-school PA [24]. Although we acknowledge the importance of perceived accessibility and/or presence of environmental attributes in PA-research, we cautiously suggest that this played a relatively minor role in our study because our playability index aggregated qualities of multiple playgrounds, accounting for accessibility and the number of these playgrounds in the school-environment.

When taking into account the relatively small strata-specific sample sizes at greater distance-categories, relationships may seem relatively weak. However, this study demonstrated that relevant relationships between physical environments and ASPA can be revealed and made plausible, with increasing specificity in time and distance. This demonstrates that loss of statistical power due to lower number of observations is compensated by increased discriminative precision thanks to time-place specificity.

Our proposed playability score allowed for aggregation of playground characteristics of the school-environment within multiple geographic settings. The SPACE observation instrument is comparable to the Neighborhood Environment Walkability Index in terms of identified factors/scales (e.g., facilities, aesthetics, proximity, accessibility), aggregation procedure (computation of means from subscale items), and normalization procedures [37, 38]. The concept of playability was introduced in the studies of Frank and Roberts [6, 41]. As the study of Roberts solely reported on a protocol of developing a playability-index, to date no direct comparisons can be made with the current study’s SPACE observation instrument. The study of Frank et al. [6] derived the quantity and quality of public parks using the Environmental Assessment of Public Recreational Spaces (EAPRS). With the exception of the quality-concept ‘shade’ and trails, all concepts and methodology of EAPRS (i.e., independent audits of two trained observers) were also represented in the current study.

One may argue that our results are not influenced by differences in playability, but by differences between schools in active transport. However, we found no indication for a ASPA-increase in children who lived more than 800 m from their school at later time-periods (potentially to compensate for motorized transport). In addition, children reported the number of days per week they walked or cycled to school and the mean duration of those trips, and sensitivity analyses revealed that additional adjusting for active transport did not alter our results (data not shown). As our sample size did not allow further segregation for active transport use, we recommend future research to address the influence of active transport in this relationship.

Our time-specific analyses showed that LPA performed after 20:00 h was significantly related to playability. This was unexpected because in the Netherlands, during this time of the year it was dark. As we had no diaries, we were unable to confirm to what extent and where children were active at that time. Apart the possibility that relationships in the late evening may be influenced by the relatively small sample sizes because of non-weartime periods, we can only speculate that that this behavior may be merely related to LPA inside their houses, or that potential differences (by chance) between schools in bed-times may have influenced this association, rather than the actual influence of playability of the environment. The same explanation may suffice for unexpected statistically significant relationships between MVPA and playability in children living more than 1600 m from their school. In addition, we observed that the MVPA percentages increased between 18:00–20:00, but strength of relationships between MVPA and playability did not increase accordingly. This potentially means that the observed increase in mean MVPA percentages was explained by other factors than playability of the environment, such as sports participation (organized forms of after-school activity often occur during evening-hours). Future studies are advised to include some diaries about sleep times and main activities after school (e.g., organized sports participation in the evenings), and are warranted to examine potential if attenuation of playability by organized sports participation would persist in spring, and whether breaks in organized sports (e.g., during summer recess) would relate to a stronger relationship between playability and PA after-school-time.

### Strengths and weaknesses

The major strength of this study is that we attempted to improve the understanding of ASPA and a potentially plausible relationship with playability of the physical environment, by measuring time-period specific ASPA and detailed, qualitative characteristics of playgrounds in school-environments.

The present study was confined to an ecological design, assessing characteristics at the school level. Recent methodological innovations such as combined accelerometry and GPS measurements can provide opportunities for even more in-depth analyses of the association between environmental attributes and domain- specific PA. GPS measurements can for example be used to identify time spent outside [26] or even at the schoolyard or at specific playgrounds (Van Kann et al. 2016, unpublished). Moreover, integrating multiple data-sources (e.g., accelerometry, GPS, GIS, audits, school’s time tables, participant diaries) into comprehensive databases provide unique opportunities for investigating PA and other health behaviors, while accounting for its spatial and temporal specificity [8, 42].

This study applied a threshold of 50 % period-specific registration time to prevent our analyses being influenced by short- spurious spikes of (intense) PA. Although two studies also used this 50 % threshold [27, 34] and one study used a 60 % threshold [26], reliability and relative influence of exact thresholds for period-specific registration times are debatable. In addition, the definition of non-weartime periods (e.g., 60 min of consecutive zero’s) highly influences period-specific thresholds, as it determines whether relatively short- or longer bouts of inactivity are classified as non-weartime periods. Future studies are therefore warranted to investigate the influence of period-specific thresholds in depth while also accounting for differences in non-weartime definitions; for example by comparing its influence in relationships with ASPA patterns.

One can speculate on alternative protocols in computing playability (e.g., multiplication of individual items or based on unequal weights). However, as we were unable to find an evidence-base for such alternative protocols, we decided to aggregate based on equal weights. To check for potential errors in the aggregation procedure we fed back the aggregated scores to the auditors of school-environments. Hereafter, no alterations were made to the aggregation protocol.

As our audits were limited to 800 m buffers from participating schools, it may seem logical that the relationship between playability of the school-environment and ASPA attenuated for children that lived outside this study-area. However, the aim of the present study was to demonstrate the temporal and site-specific mechanisms, and thus underline that in future research investigating relationships between PA and the environment, time- and place specificity is warranted. In addition, as in the Netherlands no public primary schools have organized public transport services from school to children’s homes and the majority of 8–11 children rely on active transport to get home after school, all children are likely to have at least the opportunity to be exposed to PA-opportunities in their school-environment.

As data collection was conducted in autumn, our results may not be comparable with other studies that usually perform their PA measurements in spring. Future studies are therefore encouraged to replicate this methodology in spring (or expressly study modification by season). In addition, one-third of the children in our sample experienced one hour earlier sunset due to daylight savings time change in fall. Similarly to the results of Goodman et al. [43], we found that children measured in the period with earlier sunsets were less active, both in LPA and MVPA, independent of other meteorological measures (results not shown). In addition, differences in children’s PA were especially noticeable in the evenings (data not shown). Because of the relatively unequal distribution of participants measured during daylight saving time versus standard time and our limited sample size, we were unable to check whether the association between playability and ASPA differed between children measured with daylight saving periods versus standard time.

### Impact

We found playability to be related with ASPA only in the time-period directly after-school, especially in children who live within 800 m distance from their school. First, this showed that children who lived further away from school, were relatively confined to their own residential neighborhood after-school, thus making limited use of the school-environment for ASPA. Second, playgrounds in school-environments only had a limited influence on children’s ASPA throughout the day, and competing PA-domains (e.g., sports participation) may have explained variability in especially MVPA percentages in later time-periods of the day.

## Conclusion

This study demonstrated the importance of playability of school-environments as an environmental determinant of after-school PA in children. With time and space filtering, the conceptual understanding of ASPA and its association with the physical environment can be improved. This may help to develop more tailored interventions to promote specific PA-domains at specific time-periods during the day. All in all, our analytical design with time and space filtering may encourage researchers to look into more domain-specific parts of children’s PA behavior within the opportunities and limitations of their own sample, embedded in strong theoretical foundations.

## Abbreviations

ASPA, after school physical activity; LPA, light physical activity; MVPA, moderate to vigorous physical activity; PA, physical activity.

## Appendix

**Table 5:** Associations between playability and after-school PA, stratified for time of the day and network-distance from children’s residence to school

% Light PA				
	Network-distance from children’s residence to school			
	≤ 499 m	500–799 m	800–1199 m	≥ 1200 m
End of the school day – 16:00 h (*n =* 539)	**0.060 (0.023 to 0.097)**	0.032 (−0.007 to 0.072)	**0.071 (0.038 to 0.104)**	0.002 (−0.031 to 0.036)
16:00–18:00 h (*n =* 586)	**0.040 (0.006 to 0.075)**	−0.007 (−0.042 to 0.027)	0.027 (−0.001 to 0.055)	0.013 (−0.017 to 0.042)
18:00–20:00 h (*n =* 585)	0.026 (−0.011 to 0.063)	0.015 (−0.021 to 0.051)	0.005 (−0.024 to 0.035)	**−0.040 (−0.072 to−0.009)**
20:00–22:00 h (*n =* 326)	**0.065 (0.022 to 0.108)**	0.029 (−0.014 to 0.073)	**0.067 (0.031 to 0.104)**	**0.047 (0.010 to 0.084)**
% MVPA				
	≤ 499 m	500–799 m	800–1199 m	≥ 1200 m
End of the school day – 16:00 h (*n =* 539)	**0.048 (0.007 to 0.090)**	**0.056 (0.009 to 0.102)**	**0.038 (0.003 to 0.073)**	−0.018 (−0.058 to 0.022)
16:00–18:00 h (*n =* 586)	**0.040 (0.003 to 0.077)**	**0.041 (0.003 to 0.079)**	0.020 (−0.008 to 0.048)	0.019 (−0.014 to 0.053)
18:00–20:00 h (*n =* 585)	**0.039 (0.001 to 0.078)**	0.023 (−0.016 to 0.062)	**−0.034 (−0.063 to−0.005)**	**−0.038 (−0.073 to−0.003)**
20:00–22:00 h (*n =* 326)	−0.005 (−0.052 to 0.041)	0.001 (−0.049 to 0.049)	0.027 (−0.010 to 0.064)	**0.044 (0.002 to 0.086)**

Standardized beta’s (with 95 % confidence intervals in brackets) from linear mixed model analyses with a random intercept and slope over time (one-hour periods), nested within the dates at which measurement commenced. Results were adjusted for age, gender, average temperature, average duration of rainfall, and average duration of sunshine per day. Bold number represents statistical significance at *p* <0.05
